# Development of a naringenin microemulsion as a prospective ophthalmic delivery system for the treatment of corneal neovascularization: *in vitro* and *in vivo* evaluation

**DOI:** 10.1080/10717544.2021.2021323

**Published:** 2021-12-29

**Authors:** Yu Ma, Jingjing Yang, Yali Zhang, Chunyan Zheng, Zhen Liang, Ping Lu, Fei Song, Yuwei Wang, Junjie Zhang

**Affiliations:** a Henan University of Chinese Medicine, Zhengzhou, China; b Henan Eye Institute, Henan Eye Hospital, Zhengzhou University People’s Hospital, Zhengzhou, China; c The First Affiliated Hospital of Henan University of Chinese Medicine, Zhengzhou, China

**Keywords:** Naringenin, microemulsions, ocular drug delivery, pharmacokinetics, corneal neovascularization

## Abstract

Naringenin, a flavonoid, possesses antiangiogenic potential and inhibits corneal neovascularization (CNV); however, its therapeutic use is restricted due to poor solubility and limited bioavailability. In this study, we developed a naringenin microemulsion (NAR-ME) for inhibiting CNV. NAR-ME formulation was composed of triacetin (oil phase), Cremophor RH40 (CRH40), PEG400, and water, its droplet size was 13.22 ± 0.13 nm with a narrow size distribution (0.112 ± 0.0014). The results demonstrated that NAR-ME released higher and permeated more drug than NAR suspension (NAR-Susp) in *in vitro* drug release and *ex vivo* corneal permeation study. Human corneal epithelial cells (HCECs) toxicity study showed no toxicity with NAR-ME, which is consistent with the result of ocular irritation study. NAR-ME had high bioavailability 1.45-fold, 2.15-fold, and 1.35-fold higher than NAR-Susp in the cornea, conjunctiva, and aqueous humor, respectively. Moreover, NAR-ME (0.5% NAR) presented efficacy comparable to that of dexamethasone (0.025%) in the inhibition of CNV in mice CNV model induced by alkali burning, resulting from the attenuation of corneal vascular endothelial growth factor (VEGF) and matrix metalloproteinase (MMP-14) expression. In conclusion, the optimized NAR-ME formulation demonstrated excellent physicochemical properties and good tolerance, enhanced ocular bioavailability and corneal permeability. This formulation is promising, safe, and effective for the treatment of CNV.

## 1. Introduction

Corneal transparency is essential to its optical function, and this layer is normally avascular, but under certain conditions, capillaries invade the limbal vascular plexus. Corneal disease is the third most common cause of blindness worldwide, and corneal neovascularization (CNV) is present in most affected cases. It is estimated that 1.4 million people in the USA develop CNV per year, 12% of whom suffer subsequent loss of vision (Lee et al., 1998). CNV is the main cause of vision loss worldwide (Nicholas & Mysore, 2021). The avascularity of the cornea is also due to the balance between proangiogenic stimuli and antiangiogenic factors. Numerous factors such as trauma, inflammation, infection, corneal degeneration, and hypoxia can break these homeostatic safeguards, leading to neovascularization. Some proangiogenic cytokines such as vascular endothelial growth factor (VEGF) play an important role in angiogenesis. Previous study found that the expression of VEGF shown to be higher in both animal models and human CNV than that in the normal group (Peiris-Pagès et al., 2010). VEGF-A acts as the predominant VEGF member of multiple steps in stimulating pathologic CNV. Matrix metalloproteinases (MMPs) are also required for corneal angiogenesis (Han et al., 2016).

Topical steroids remain a first-choice treatment for CNV (Liarakos et al., 2014). However, the long-term use of steroids causes various serious side effects, which has promoted a search for an effective alternative. Antiangiogenic therapies have been demonstrated to have limited or partial effects in reducing pathological CNV in animal studies and clinical trials (Chung & Ferrara, 2011). Thus, further study is needed to search for safer and more effective therapies for CNV. Recently, NAR has been reported to have an anti-CNV function. NAR is a type of flavanone that is widely derived from natural Rutaceae plants and is the most abundant in grapefruit. NAR has attracted the attention of many researchers because it presents a wide variety of interesting pharmacological effects, including anticancer, insulin-like, antioxidant, anti-inflammatory, antiproliferative, antifibrogenic, neuroprotective, antidiabetic, and immunomodulatory effects (Bhia et al., 2021). Recent studies have demonstrated that NAR has a promising degree of efficacy in treating eye disorders, such as inhibiting CNV (Oguido et al., 2017), exhibiting an anti-inflammatory effect on uveitis in rats (Shiratori et al., 2005), improving neurotrophic effects in the diabetic rat retina (Al-Dosari et al., 2017), promoting protective effects for photoreceptor cells in rats (Lin et al., 2014), and inducing anti-choroidal neovascularization in rats. However, topical ocular drug delivery of NAR for the treatment of eye disorders is greatly limited due to its poor aqueous solubility. NAR-encapsulated sulfobutylether-β-cyclodextrin/chitosan nanoparticles (Zhang et al., 2016), hydroxypropyl-β-cyclodextrin/NAR complexes (Lin et al., 2015), and nanocomplexes of NAR-based polyvinylpyrrolidone (Wang et al., 2020) have been developed for ocular drug delivery to improve the solubility and progress; however, some important parameters such as storage stability and therapeutic efficacy were not addressed in these studies.

NAR is poorly water-soluble owing to its hydrophobicity, which limits its clinical application. MEs are among the most promising nanocarriers for drug delivery, especially poorly water-soluble drugs. ME formulations usually significantly increase the capacity for loading insoluble water drugs. MEs as ocular drug delivery systems offer many advantages over conventional eye drops, including increased ocular retention, possibility of releasing drugs in sustained and controlled ways, effective penetrating ocular barriers, improved bioavailability, accurate dosing, and increased shelf life. The aim of this work was to develop NAR microemulsions (NAR-MEs), optimize the formulations by using central composite design response surface methodology (CCD-RSM) via the design of experiments (DoEs) approach, and evaluate both the *in vitro/in vivo* performance of NAR-ME as a topical ophthalmic delivery system for the treatment of CNV in a mouse model and ocular pharmacokinetics in rabbits.

## 2. Materials and methods

### 2.1. Materials

NAR was purchased from Macklin Biochemical Co., Ltd. (Shanghai, China), glycerol triacetate was bought from J&K Scientific Ltd. (Beijing, China), oleic acid was obtained from Tokyo Chemical Industries Co., Ltd. (Tokyo, Japan), propylene glycol dicaprylate and castor oil were purchased from Hunan Er-Kang Pharmaceutical Co., Ltd. (Changsha, China), PEG-40 hydrogenated castor oil (Cremophor RH40, CRH40), Cremophor EL35, and Kolliphor HS15 were bought from BASF SE (Ludwigshafen, Germany). Tween 80 was purchased from Sichuan Jinshan Pharmaceutical Co., Ltd. (Guangyuan, China), PEG400 was obtained from Solarbio Life Science (Beijing, China), and dexamethasone (DXMS) sodium phosphate eye drops were obtained from Xinxiang Huaqing Pharmaceutical Co., Ltd. (Xinxiang, China). The other reagents and solvents were analytical grade and high-performance liquid chromatography (HPLC) grade, respectively.

### 2.2. Animals

Forty-two New Zealand white rabbits weighing 2.0–2.5 kg were obtained from Huaxing Experimental Animal Farm (Zhengzhou, China). Eighty 6- to 8-week-old male BALB/c mice weighing 18–22 g were purchased from the same place. All animals had free access to a standard diet and drinking water and were housed in a room maintained at 22.0 °C ± 3 °C and with a 12:12 h cyclic lighting schedule. All animal experiments conformed with the principal of Association for Research in Vision and Ophthalmology (ARVO) statements. All animal procedures were approved by the Experimental Animal Ethics Committee of Henan Institute of Ophthalmology.

### 2.3. HPLC analysis of NAR

The NAR content was assayed by HPLC (Waters 2695 liquid chromatography system, Milford, MA) using a Waters XBridge^®^ C18 column (4.6 mm × 150 mm, 5 μm) with a column temperature of 40 °C. The mobile phase consisted of a mixture of water and methanol (40:60, v:v) with a flow rate of 1.2 mL/min. Detection was performed at a wavelength of 288 nm, and the injection volume was 10 μL. The NAR concentration was obtained from a standard curve. Stock solutions of NAR were prepared by dissolving accurately weighed standard NAR in methanol, and serially diluted working solutions were obtained through stepwise dilutions of the stock solution with the mobile phase. Then, the standard curve was obtained.

### 2.4. Screening of oil, surfactant, and cosurfactant

The saturation solubilities of NAR were determined in oils (olive oil, oleic acid, propylene glycol dicaprylate, castor oil, and triacetin), surfactants (Cremophor EL35, CRH40, Tween 80, and Kolliphor HS15), and cosurfactant (PEG400). Excess NAR was added to 1 g of each oil, surfactant, and cosurfactant in closed glass vials and vortexed in a shaking water bath at 100 rpm at 37 °C for 48 h. After equilibrium, the mixtures were centrifuged at 10,000 rpm for 30 min, and the clear supernatant liquid was separated. The quantity of NAR in the supernatant after dilution with ethanol was determined by HPLC (Section 2.3). All experiments were conducted in triplicate, and NAR solubility (mg/g) in each vehicle was recorded as the mean value ± standard deviation (SD) (Shao et al., 2021). The components with the high solubility to NAR were selected to construct the pseudoternary phase diagram.

### 2.5. Construction of pseudoternary phase diagrams

Distilled water was added dropwise to a mixture of oil and S/Cos (Km) under gentle magnetic stirring at 37 °C until the mixture became clear at a certain point (Kassaee & Mahboobian, 2021; Zeng et al., 2021). The system with low viscosity and a clear appearance was considered to be a microemulsion. The critical points between the ME region and other phase regions were determined when the appearance of the system changed from clear to turbid and vice versa. The amount of water consumption was recorded and used to calculate the final weight percentages of water, oil, and Km in order to complete the pseudoternary phase diagrams. Finally, Origin Pro software (Version 9.1, Northampton, MA) was used to construct the pseudoternary phase diagrams.

### 2.6. Optimization of NAR-ME by central composite design-response surface methodology

To optimize the various variables of the formulation, Design-Expert^®^ software (Stat-Ease, Inc., Minneapolis, MN) for the systematic DoEs was employed. The critical independent variables (*X*1: oil weight; *X*2: Km weight) were subjected to the optimization phase by employing central composite design-response surface methodology (Zhu et al., 2008). The investigated dependent responses were the droplet size (DS) of the prepared MEs (*Y*1) and drug loading (DL) (*Y*2). The independent factors and determined responses are shown in Table 1.

**Table 1.** Central composite design-response surface methodology used for optimization of NAR-ME formulae.

Factors (independent variables)	Levels						
	–1.414	–1	0	1		1.414	
*X*1 (oil weight/g)	0.175736	0.3	0.6		0.9	1.02426	
*X*2 (Km weight/g)	1.97574	2.1	2.4		2.7	2.82426	

Responses (dependent variables)	Desirability constraints
*Y*1: droplet size (DS/nm)	Minimize
*Y*2: drug loading (DL/%)	Maximize

### 2.7. Preparation of NAR-ME

From the screen results of central composite design-response surface methodology, the optimal prescription of the NAR-ME was selected. NAR-loaded formulations were prepared by a spontaneous emulsion method. Briefly, the oil phase (triacetin), surfactants, and cosurfactant were mixed well by magnetic stirring at 37 °C. NAR was dissolved in the mixture. Finally, distilled water was added dropwise to the mixture, followed by vortexing for 1–2 min to obtain a homogeneous mixture. No precipitate was observed in the final drug-loaded formulations.

### 2.8. Thermodynamic stability studies

The transparency of NAR-ME was determined by visual inspection, and the thermodynamic stability of NAR-ME was assessed in three stages. First, NAR-MEs were exposed to three heating (40 °C) and cooling (4 °C) cycles by storage at each temperature for 48 h. Furthermore, samples were submitted to three freezing (–20 °C) and thawing (25 °C) cycles with storage at each temperature for two days, and finally, they were centrifuged at 10,000 rpm for 30 min (Gorain et al., 2014).

### 2.9. Storage stability

The optimized NAR-ME formulations were prepared and stored in light-protected and tightly sealed glass bottles at 4 °C, 25 °C, and 40 °C for three months. After 1, 2, and 3 months of storage, both the physical and chemical stabilities were assessed. The physical stability evaluations of NAR-ME included appearance, DS, polydispersity index (PDI), and pH. The chemical stability was evaluated by determining the remaining NAR content using HPLC. The percentage of NAR remaining was compared with the amount on day 0 (baseline). The samples at each time point were assayed in triplicate.

### 2.10. Characterization of NAR-ME

#### 2.10.1. Determination of droplet size, polydispersity index, and zeta potential

The average DS, PDI, and zeta potential (ZP) of NAR-ME formulations were determined using dynamic light scattering (DLS) (Zetasizer, NanoZS90, Malvern Instruments, Worcestershire, UK), where light scattering was monitored at 25 °C and an angle of 90°. All samples were measured without any dilution and analyzed in triplicate.

#### 2.10.2. Drug content and encapsulation efficiency determination

The NAR-ME samples were diluted with an appropriate amount of mobile phase, and the NAR content was determined with a previously developed HPLC method (Section 2.3) for triplicate. The encapsulation efficiency (EE) and DL of NAR-ME were calculated as follows:

(1) $$\mathrm{EE}\;(\%)=\frac{\text{weight of the drug in the ME }}{\text{weight of the drug added}}\;\times \;100\%$$

(2) $$\mathrm{DL}\;(\%)=\frac{\text{weight of the drug in the ME }}{\text{weight of the drug and nanocarrier}}\;\times \;100\%$$

#### 2.10.3. Morphological examination

The morphology examination of the optimized NAR-ME was carried out using transmission electron microscopy (TEM) (Joel JEM 1230, Tokyo, Japan). Briefly, a drop of diluted NAR-ME sample was placed onto a carbon-coated copper grid, and the excess sample volume was removed with filter paper. The sample was then negatively stained by a drop of 2% phosphotungstic acid solution, and the surface of the carbon grid was air-dried at room temperature before being loaded into the TEM system for testing.

#### 2.10.4. pH and osmotic pressure

The pH values of NAR-MEs were determined at 25 °C using a calibrated pH meter (Leici, Shanghai, China). The osmotic pressure of the NAR-ME solution was determined by a freezing point osmometer (STY-1A, Tianjin, China). One hundred microliters of NAR-ME solution was used for osmotic pressure determination. All measurements were carried out in triplicate.

#### 2.10.5. *In vitro* drug release from NAR-MEs

The *in vitro* release of NAR from NAR-MEs was performed by the dialysis method in simulated tear fluid (STF) (NaHCO_3_ 0.218 g, NaCl 0.678 g, CaCl_2_ 0.0063 g, KCl 0.138 g in 100 mL of purified water) using a dialysis bag (molecular weight cutoff of 8000–14,000) at 37 °C. An aliquot of 0.5 mL of each 1% of NAR-ME (*W*_0_=5 mg) was placed into dialysis bags and dialyzed against the release medium at 37 °C ± 0.2 °C in an air bath shaker (Changzhou Putian Instrument Manufacturing Co. Ltd., Changzhou, China) at 100 rpm. At predetermined intervals (0.25 h, 0.5 h, 1 h, 2 h, 4 h, 6 h, 8 h, 10 h, 12 h, 24 h, 36 h, 48 h, and 72 h), 1.0 mL of the release medium was withdrawn, and an equal volume of fresh STF was added to maintain sink conditions. The drug concentration was determined by using a UV spectrophotometer (UV1800SPC, Shanghai Meixi Instrument Manufacturing Co. Ltd., Shanghai, China) at a wavelength of 288 nm, and the accumulative release percentage (*Q*) of NAR released from NAR-ME was calculated by Equation (3). The cumulative release profile was plotted by using Origin Pro software (Version 9.1, Northampton, MA). The same amount of NAR suspension (NAR-Susp) in STF was chosen as a control. One percent of NAR-Susp was obtained by adding 0.5 g of NAR to 50 mL of 0.5% of hydroxymethyl cellulose and holding it for at least 20 min in an ultrasonic bath. The particle size was determined to be <25 μm by light microscopy in accordance with the Chinese Pharmacopeia. These experiments were carried out in triplicate for every sample. For kinetic analysis, the release data were fitted to various kinetic models such as zero order, first order, and Higuchi’s equation.

(3) $$Q(\%)=\frac{C_{n}V+V_{i}\sum_{i=0}^{i=n}C_{i}}{W_{0}}\times 100\%$$

where *W*_0_ is the total weight (5 mg) of the drug in NAR-ME added in the dialysis bag, *C_n_* is the NAR concentration in the release medium at *t_n_*, *V* is the total volume of release medium (100 mL), *V_i_* is the sample volume (1.0 mL) at *t_i_*, *C_i_* is the sample concentration at *t_i_*, and *t_n_* is the sampling at the *n*th time (Li et al., 2016).

### 2.11. *In vitro* human corneal epithelial cytotoxicity

The cytotoxic effects of NAR-ME were evaluated via a Cell Counting Kit-8 (CCK-8) assay (APExBIO, Houston, TX). The human corneal epithelial cells (HCECs) were purchased from APExBIO Technology LLC (Beijing, China). The culture medium used was Dulbecco’s modified Eagle’s medium (DMEM). HCECs suspension (100 μL) was seeded into a 96-well plate at a density of 5000 cells/well. After incubation for 24 h, the medium was discarded, and 100 μL of NAR-ME and 100 μL of blank ME with different concentrations were added (the ratios of NAR-ME or blank ME to the medium were 1:1, 1:10, 1:50, and 1:100). Growth medium without NAR-ME was used as a control. After being cultured for 15 min, 1 h, 2, and 4 h in a 5% CO_2_ atmosphere at 37 °C, HCECs were washed with phosphate-buffered saline (PBS, pH 7.4) three times and incubated with 10 μL of 10% CCK-8 solution per well for another 3 h. Absorbance was measured in a microplate reader spectrophotometer at 450 nm (PerkinElmer 2104 Multilabel Reader, Waltham, MA). The percentage of cell growth inhibition was calculated by the following equation:

(4) $$\text{Control of cell growth }(\%)=100-\frac{\text{mean OD of individual test group}}{\text{mean OD of control group}}\times 100$$

### 2.12. Ocular irritation test

Ocular irritation tests with the formulation were performed in six New Zealand White rabbits according to the modified Draize test (Wilhelmus, 2001). Before the test, both eyes of each rabbit were checked and verified to have no lesions or inflammation. One single dose of 100 μL ophthalmic NAR-ME was instilled the lower conjunctival cul-de-sac of left eye. After instillation, the eyelid was closed manually for 30 s to prevent the loss of the instilled solution. The right eye served as a control and was treated with saline. Signs of ocular irritation such as redness, discharge, and chemosis of the conjunctiva as well as the status of the cornea and iris were examined and scored in both eyes of the rabbit pre-exposure and post-exposure at different time points (1, 2, 4, 24, 48, and 72 h) pre- and postexposure. The irritation score was the mean value of the sum score from six treated eyes, and the irritation level was evaluated according to the Draize technique. According to the observed irritation, the score at each time was given in the range of 0–3 for no irritation. A sum score (entire clinical evaluation) of more than 4 in total for the ocular irritation index or a score of 2 or 3 in any item indicated a significant irritant (Kalam et al., 2016).

### 2.13. *Ex vivo* corneal permeation study

An *ex vivo* corneal permeation study was performed on corneas isolated from rabbit whole eyes using side-by-side diffusion cells with a permeation area of 0.694 cm^2^ following a previous report method (Baspinar et al., 2008; Alkholief et al., 2019). Briefly, freshly excised corneas from New Zealand albino rabbits were mounted between the donor and receptor chambers of side-by-side diffusion cells such that the corneal side remained in contact with the formulations. Five milliliters of STF (pH = 7.4) was filled in the thermostated (34 °C ± 1 °C) receptor chamber and donor chambers under 95%:5% (O_2_:CO_2_) aeration and preincubated for 30 min. The fluid in receptor compartment was on magnetic stirring (500 rpm) for removal of air bubbles throughout the study. Two milliliters of STF was removed, and then, an equal volume of 1% of NAR-ME eye drops was added to the donor chambers. At predetermined intervals (15, 30, 60, 90, 120, 180, and 240 min), 200 μL of samples was collected from the receptor chamber and replenished immediately with an equal volume of fresh STF at each time interval. The withdrawn samples were analyzed for drug content by HPLC. The same amount of NAR-Susp in the donor chamber was chosen as a control. The experiments were carried out in triplicate. At the end of the permeation study, the scleral ring around the cornea was cautiously excised and removed; then, the cornea was rinsed with water, and excess water was removed using a paper filter. The wet weight (*W_a_*) of each cornea sample was then measured. Corneas were dried in an incubator at 70 °C for 12 h to obtain the dry corneal weight (*W_b_*). Equation (5) was used to calculate the percentage of corneal hydration level (HL) for corneas treated with the formulation (Liu et al., 2011):

(5) $$\mathrm{HL}\;（\%）=\frac{W_{a}-W_{b}}{W_{a}}\;\times 100\%$$

The permeation parameters of NAR from ME were calculated by plotting the amounts of drug permeated through the cornea (μg/cm^2^) versus time (h), and the slope of the linear portion of the graph was calculated. The cumulative penetration (*Q_n_*) was calculated by using Equation (6), the steady-state flux (*J*) values across the cornea were calculated by using Equation (7), and the coefficient of permeation (*P_app_*) was evaluated by Equation (8) from the linear ascents of the permeation plots:

(6) $$Q_{n}\;({\mu g/\mathrm{cm}}^{2})=(C_{0}V_{0}+V\sum_{i=1}^{n-1}C_{i})/A$$

(7) $$J\;({\mu g/\mathrm{cm}}^{2}/s)=\frac{dQ}{dt\times 3600}$$

(8) $$\;P_{\mathrm{app}}\;(\mathrm{cm}/s)=\frac{J}{C_{0}}$$

where *C*_0_ represents the initial drug concentration (4000 μg/mL) in the donor compartment. *V*_0_ is the total volume (5 mL) in the donor compartment, *V* is the sample volume (0.2 mL), *C_i_* is the sample concentration at a specific sampling point in time, *J* is the linear portion of the slope, *A* is the exposed area of the cornea (0.694 cm^2^), 3600 is the conversion of units from hours to seconds, and *t* is the exposure time.

Finally, the HL of each cornea used for this experiment was calculated at the end of the experiment.

### 2.14. Ocular pharmacokinetic studies in rabbits

#### 2.14.1. Rabbits and treatments

Forty-two healthy New Zealand rabbits (weighing 2.0–2.5 kg) with no eye diseases were randomly divided into an experimental group and a control group (*n* = 21 in each group). The NAR-ME solution was administered to the bilateral eyes of each animal in the experimental group, while animals in the control group were administered NAR-Susp. Both groups were randomly divided into seven subgroups according to the predetermined time points after dosing (*n* = 3 in each group). One single 50 μL dose of the NAR-ME solution or NAR-Susp was instilled into the lower eye cul-de-sac (both eyes), and the eyelids were kept closed manually for 30 s after administration. At predetermined time points after dosing (5, 10, 15, 30, 60, 90, and 120 min), tear fluids were collected using preweighed filter paper disks, and the animals were euthanized via an injection of a lethal overdose of pentobarbital sodium into the ear vein. Aqueous humor (100 μL) was withdrawn, corneal and conjunctival tissues were collected and rinsed with saline, blotted using filter paper and weighed. All samples were stored at −80 °C until analysis.

#### 2.14.2. Analysis of ocular tissues, aqueous humor, and tear fluids

A validated method reported by Zhang et al. (2016) was modified to extract NAR from rabbit cornea, conjunctiva, aqueous humor, and tear fluids. Briefly, 400 μL of methanol was added to each aliquot of cornea and conjunctiva tissues to immerse for 24 h with sealing, 100 μL of methanol was mixed with aqueous humor, and 500 μL of methanol was added to tear samples and vortexed for 1 min. All methanol mixture samples were centrifuged at 10,000 rpm for 10 min (MiniSpin^®^ Plus, Eppendorf, Hamburg, Germany), and the supernatants were used for HPLC analysis.

Analytical method validation was validated for specificity, linearity, recovery, precision, and accuracy, and stability according to the U.S. Food and Drug Administration guidelines for the validation of bioanalytical methods and successfully applied to assay the levels of NAR in the tissue samples for the pharmacokinetics study (Supplementary Figs. S1, S2, and Tables S1, S2).

### 2.15. *In vivo* anti-corneal neovascularization efficacy

#### 2.15.1. Establishment of a corneal neovascularization mouse model

Corneal neovascularization of mice was induced using an alkali burn injury method (Irani et al., 2017). All procedures and tests were performed under general anesthesia by intraperitoneal injections of 1% pentobarbital sodium (80 mg/kg) and topical administrations of 0.4% oxybuprocaine hydrochloride eye drops. Alkali burn injury to the right eye’s cornea was induced by placing sterilized filter paper (2 mm in diameter) soaked with 2 μL of NaOH solution (1 M) for 20 s (Liu et al., 2019; Sun et al., 2019). Then, the ocular surface and conjunctival fornixes were extensively flushed with 20 mL of sterile saline solution. One day after alkali burn injury, CNVs were imaged under a slit lamp microscope (SLM-8E, Chongqing Kanghua, Chongqing, China). Subsequently, the CNV mice were randomly divided into five groups, with 16 subjects in each group. The first group received 5 μL of saline as eye drops, the second group received 5 μL of 0.25% NAR-ME (L), the third group received 5 μL of 0.5% NAR-ME (M), the fourth group received 5 μL of 1% NAR-ME (H), and the fifth group received 5 μL of DXMS eye drops. All groups were treated four times a day for seven days.

#### 2.15.2. Assessment of corneal neovascularization

Alkali-burned eyes in all groups were observed on day 1, day 3, and day 7 after injury, and corneal epithelial damage in each group was estimated via a slit-lamp microscope digital system.

To evaluate the development of CNV, the quantification of CNV was conducted by a flat-mount-based method (Li et al., 2016; Irani et al., 2017). Briefly, on day 7, three animals from each group were injected with 4 units of heparin (Zhuhai Beso Bio Co., Ltd., Zhuhai, China) per 10 g of body weight by intraperitoneal injection and sacrificed by an overdose of pentobarbital sodium solution. Hematoxylin solution (1:3 hematoxylin:PBS, pH 6.0) perfusion via the aorta was performed. The eyes were fixed overnight in 10% formalin at 4 °C, and then, the fixed cornea was removed and flattened for image capture. Quantitative measurements of CNV length and area were assessed using ImageJ analysis software (version 1.41o, National Institutes of Health, Bethesda, MD).

#### 2.15.3. Histopathological examination

Mice were euthanized on day 7 after alkali burn injury. Three mice from each group were sacrificed, and whole eyeballs were harvested. The corneas were fixed in 4% paraformaldehyde after trimmed for 24 h at 4 °C, and then embedded in paraffin wax and cut into 5 μm sections for hematoxylin–eosin (H&E) histopathological observation.

#### 2.15.4. Enzyme-linked immunosorbent assay (ELISA)

Corneal tissues were collected at 3 d and 7 d after alkali burn injury. Each time point of one group consisted of five mice. Cornea tissue samples were weighed and immediately frozen at −80 °C until analysis. When preparing the corneal tissue homogenate, it was removed from the freezer at −80 °C and rewarmed at 4 °C for half an hour. The cornea was cut into small pieces with ophthalmic tissue scissors, 100 μL of RIPA was added to submerge the tissue, which was then chilled in an ice bath for 1.5 h and centrifuged at 12,000 rpm at 4 °C for 5 min, and the supernatant was transferred to another eppendorf (EP) tube for later use. The protein levels of VEGF-A and MMP14 in the cornea were normalized to the total protein content, as determined by a bicinchoninic acid (BCA) kit (Solarbio, Beijing, China), following the manufacturer’s instructions. Absorbance at 450 nm was measured by a microplate reader (PerkinElmer 2104 Multilabel Reader, Shanghai, China).

### 2.16. Statistical data analysis

All data were analyzed using SPSS software (SPSS version 26, Chicago, IL). An independent-samples *t*-test was used to analyze and compare the NAR-ME and NAR-Susp *in vivo* ocular pharmacokinetic studies. Multiple comparisons in ANOVA were used. Fisher’s least significant difference (LSD) test was conducted to compare the differences among individual groups. *p*< .05 was considered statistically significant. The results are presented as the mean ± standard error of mean (SEM) or mean ± SD.

## 3. Results

### 3.1. Screening of oil, surfactant, and cosurfactant

The solubility values of NAR in various vehicles are listed in Table 2, where the highest solubility of NAR was obtained in triacetin (oil), CRH40 (surfactant), and PEG400 (co-surfactant). Therefore, these components were selected for ME development in the next stage.

**Table 2.** Solubility value of NAR in various vehicles.

Vehicle	Component	Solubility (mg/g)
Oil	Olive oil	0.07 ± 0.03
	Oleic acid	0.63 ± 0.03
	Propylene glycol dicaprylate	6.90 ± 0.84
	Castor oil	7.50 ± 1.79
	Triacetin	46.24 ± 0.39
Surfactant	Solutol^®^HS15	76.28 ± 3.51
	Tween 80	82.83 ± 1.20
	Kolliphor^®^EL35	83.33 ± 6.46
	Cremophor^®^RH 40	103.21 ± 2.87
Co-surfactant	Polyethylene glycol 400 (PEG400)	263.88 ± 16.36

Data represented as mean ± SD, *n* = 3.

### 3.2. Construction of pseudoternary phase diagrams

**Figure 1.**
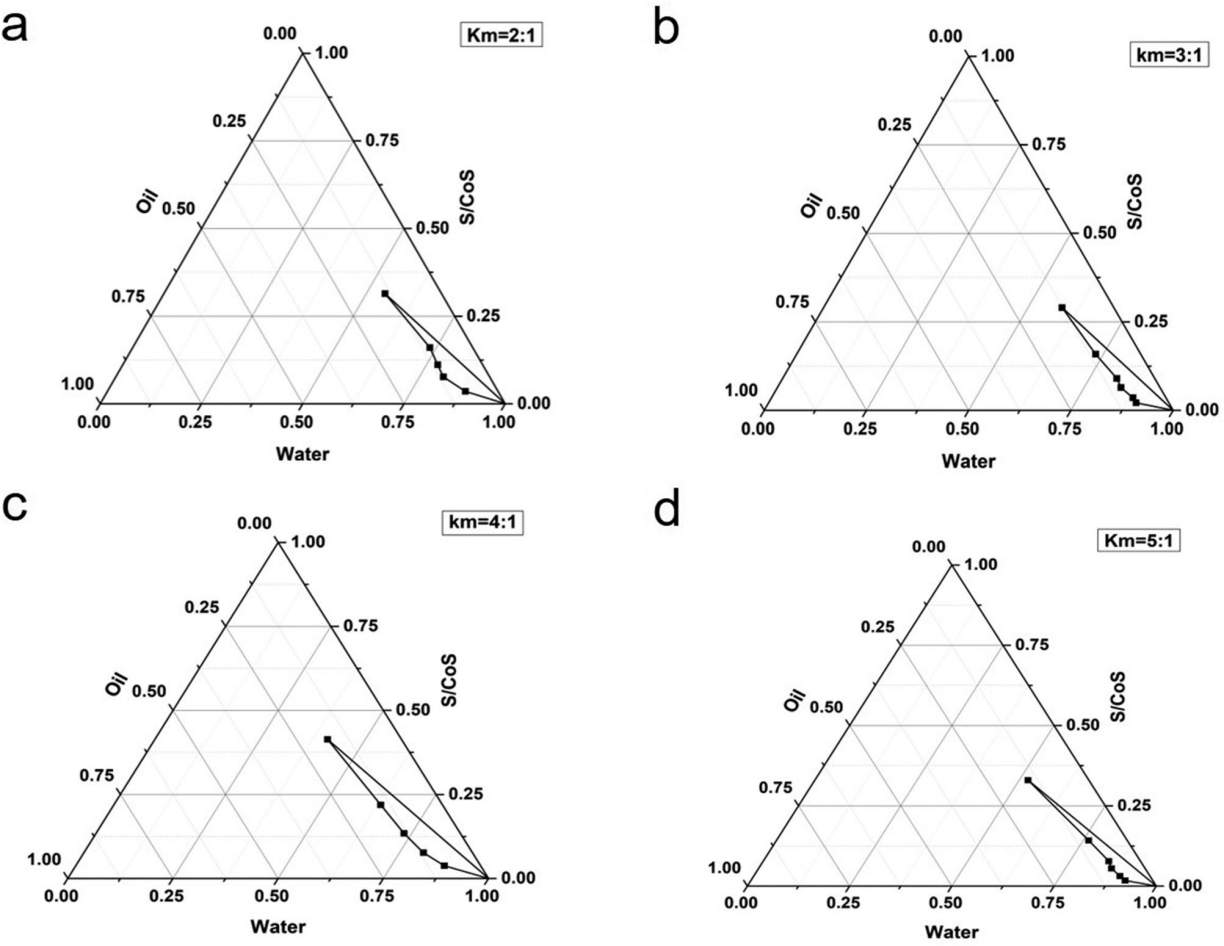
Partial pseudo-ternary phase diagrams of systems with different Km: (a) Km = 2:1; (b) Km = 3:1; (c) Km= 4:1; (d) Km = 5:1.

The concentration of each constituent plays a significant role in the formation of a stable ME; consequently, four pseudoternary phase diagrams were obtained by the water titration method at different Km values of 2:1, 3:1, 4:1, and 5:1. As illustrated in Figure 1, all four systems demonstrated the O/W ME region, and the most extensive ME region was Km = 4:1. Thus, the ratio of surfactant and cosurfactant was 4:1 for further studies.

### 3.3. Optimization of NAR-ME by the central composite design-response surface methodology

We used the Design-expert program to obtain the optimal formulation of NAR-ME using central composite design-response surface methodology. The developed design suggested 13 experimental runs representing all possible combinations of the different levels of the studied factors (Table 3).

**Table 3.** Composition and observed responses in CCD-RSM for NAR-ME.

No.	Actual value		Value of response	
	*X*1 (g)	*X*2 (g)	*Y*1 (nm)	*Y*2 (%)
1	0.3	2.7	13.61	2.660
2	0.175736	2.4	13.75	2.835
3	0.6	2.4	17.20	2.940
4	0.3	2.1	13.93	2.790
5	0.9	2.7	13.42	2.595
6	0.6	2.4	17.20	2.940
7	0.9	2.1	24.99	2.115
8	0.6	1.97574	21.49	2.770
9	0.6	2.4	17.20	2.940
10	0.6	2.4	17.20	2.940
11	0.6	2.82426	13.28	2.825
12	0.6	2.4	17.20	2.940
13	1.02426	2.4	29.46	2.095

*X*1: oil; *X*2: Km; *Y*1: droplet size; *Y*2: drug loading.

The mathematical model describing the relationship between various A (oil (g)) and B (Km (g)) values and DS (*Y*1) is summarized in the following equation:

$$\text{Droplet}\;\text{size}\;\left(Y1\right)=17.20+5.55A-2.90B-2.81AB+1.45A^{2}-0.6594B^{2}-0.0698A^{2}B-2.84AB^{2};\;R^{2}=0.9379$$

The mathematical model describing the relationship between various A (oil (g)) and B (Km (g)) values and DL (*Y*2) is summarized in the following equation:

$$\text{Drug}\;\text{loading}\;\left(Y2\right)=2.94-0.2233A+0.0535B+0.1525AB-0.2603A^{2}-0.0941B^{2};\;R^{2}=0.9641$$

**Figure 2.**
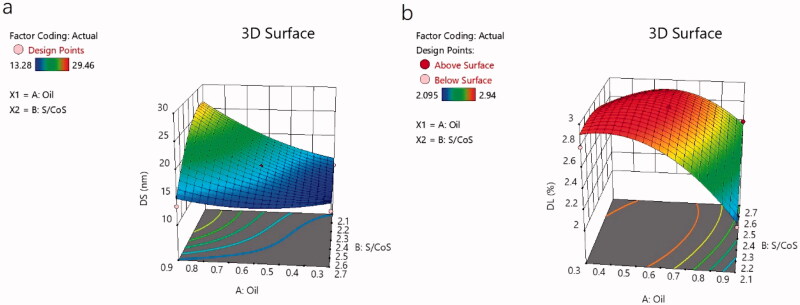
3D Response surface plot showing the effect of independent variables: (a) 3D response surface plot showing the effect of independent variables on droplet size (*Y*1); (b) 3D response surface plot showing the effect of independent variables on drug loading (*Y*2).

The effect of these variables on the DS and DL of NAR-ME can be demonstrated by three-dimensional response surface curves (Figure 2).

### 3.4. Preparation and characterization of NAR-ME

According to the above-mentioned procedures, we found that when the ratios of oil (triacetin) to Km (CRH40:PEG400 = 4:1, w/w) were fixed at approximately 1:4.35 (w/w), 10 mg/mL NAR was soluble and could be incorporated into the final ME system, and the optical appearance of the formulation was transparent, with excellent physical stability.

**Figure 3.**
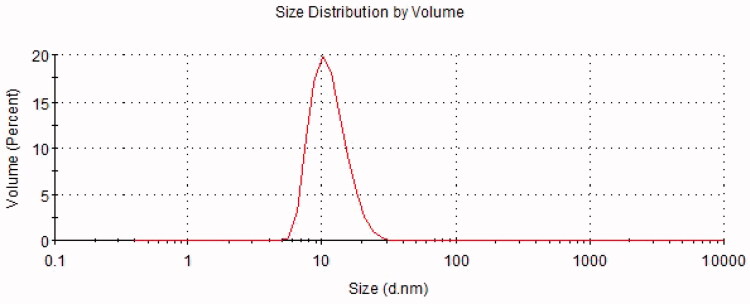
Size distribution of NAR-ME.

**Figure 4.**
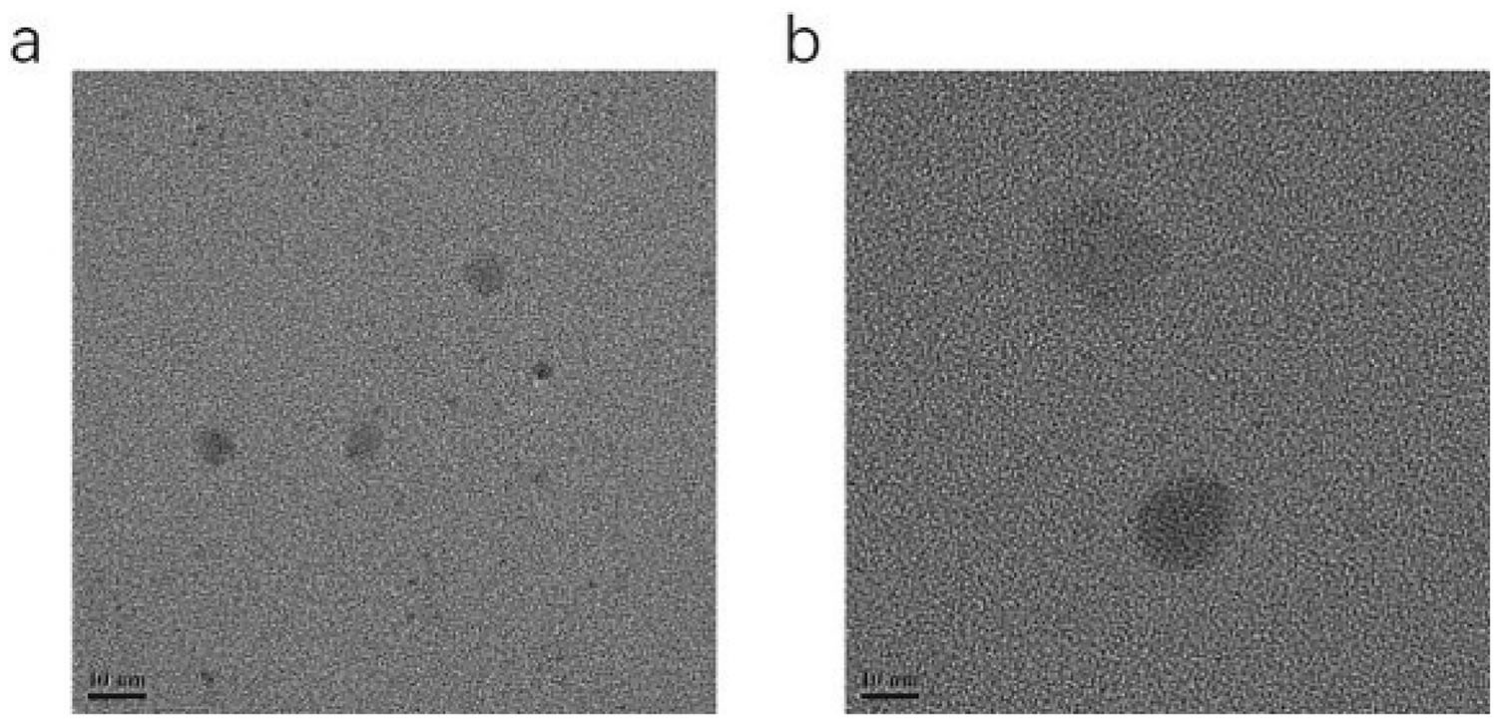
Transmission electron microscope image: (a) TEM image of optimized blank-ME; (b) TEM image of optimized NAR-ME. Scale bar = 10 nm.

The results of the pH, osmolarity, EE, DL, ZP, average DS, and PDI are summarized in Table 4. A representative graph of the average size is depicted in Figure 3, indicating that the particle size distribution by intensity was narrower, which enhanced the stability of the ME system by resisting gravitational separation. The smaller PDI value indicated that the formulation has a narrow distribution in size. The TEM image of NAR-ME (Figure 4(b)) demonstrated the spherical and uniform shape of the ME with a small DS, which was in agreement with the DS distribution data. The pH and osmolarity values were close to the normal physiological levels of the human eye, which indicates that the formulation is suitable.

**Table 4.** Physicochemical characteristics of optimized NAR-ME.

pH	Osmolarity (mmol/L)	EE (%)	DL (%)	DS (nm)	ZP (mv)	PDI
6.06 ± 0.12	307.67 ± 3.79	99.63 ± 0.03	2.92 ± 0.00	13.22 ± 0.13	1.43 ± 0.37	0.112 ± 0.001

EE: encapsulation efficiency; DL: drug loading; DS: droplet size; ZP: zeta potential; PDI: polydispersity index.

Data represented as mean ± SD, *n* = 3.

**Figure 5.**
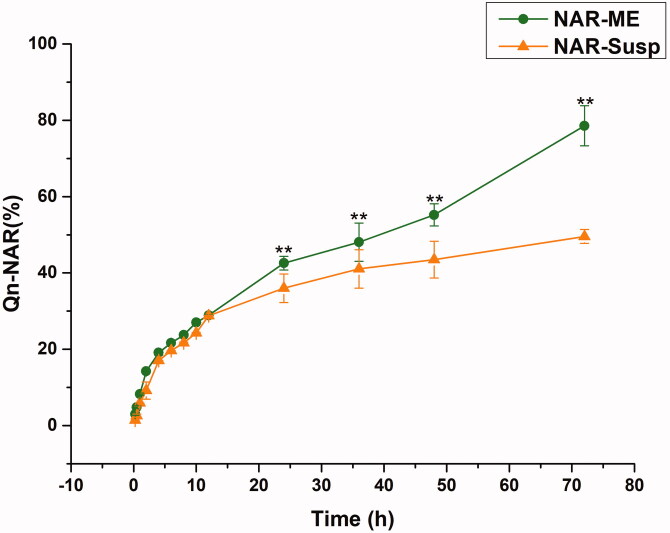
Comparative drug release profile of optimized ME and Susp solution. Data represented as mean ± SEM, *n* = 3. **p<.05. Independent samples *t*-test.

The drug release profiles of NAR-ME and NAR-Susp in STF are depicted in Figure 5. Almost 80% of NAR was released from the ME within 72 h, and NAR-ME had a better sustained release property at the later time points. However, less than 40% of NAR was released from the Susp within 72 h, indicating that the ME increased NAR release. Three kinetic models were utilized to investigate the release mechanism of NAR-ME and NAR-Susp. As shown in Table 5, the Korsmeyer-Peppas model was the best fit for the NAR-ME release profile, and the Higuchi model was the best fit for the NAR-Susp release profile.

**Table 5.** Correlation coefficients (*R*^2^) and constant values for the different mathematical models applied to the release of NAR.

Formulae	Mathematical models									
	Zero order		First order		Higuchi			Korsmeyer-Peppas		
	*k* (/min)	*R* ^2^	*k* (/min)	*R* ^2^	*k* ($/\sqrt{\text{min}}$)		*R* ^2^	*k* (/min)	*n*	*R* ^2^
NAR ME	0.94	0.90	–0.07	0.87	8.61		0.98	7.08	0.55	0.99
NAR Susp	0.64	0.79	–0.08	0.97	6.16	0.96		6.04	0.54	0.93

### 3.5. Thermodynamic stability studies

To test the thermodynamic stability of the developed formulations, it was critical to assess the capability of these formulations to cope with thermomechanical stress tests. In the present study, MEs were stable under thermal and mechanical stress, which may be correlated with the imposed repulsion. These formulations did not show any signs of instability (phase separation, turbidity, nucleation for crystal growth, and precipitation). The test results indicate a long-term shelf life of these MEs compared to conventional emulsions.

**Table 6.** Results of stability testing of NAR-ME.

Temperature	Characteristics	Time			
		0 M	1 M	2 M	3 M
4 °C	Content (mg/mL)	9.86 ± 0.03	9.82 ± 0.02	9.80 ± 0.01	9.79 ± 0.02
	Droplet size (nm)	13.08 ± 0.12	14.03 ± 0.23	13.31 ± 0.16	13.11 ± 0.13
	PDI	0.126 ± 0.00	0.106 ± 0.00	0.024 ± 0.00	0.037 ± 0.00
	pH	5.97 ± 0.02	5.70 ± 0.10	5.63 ± 0.12	5.52 ± 0.16
25 °C	Content (mg/mL)	10.09 ± 0.01	10.00 ± 0.01	10.00 ± 0.02	9.99 ± 0.01
	Droplet size (nm)	13.33 ± 0.12	13.97 ± 0.26	12.94 ± 0.11	12.97 ± 0.15
	PDI	0.112 ± 0.00	0.101 ± 0.00	0.036 ± 0.00	0.048 ± 0.00
	pH	6.02 ± 0.13	5.97 ± 0.05	5.93 ± 0.11	5.87 ± 0.12
40 °C	Content (mg/mL)	9.83 ± 0.03	9.81 ± 0.03	9.76 ± 0.01	9.71 ± 0.02
	Droplet size (nm)	13.242 ± 0.11	13.98 ± 0.23	13.01 ± 0.13	12.97 ± 0.21
	PDI	0.098 ± 0.00	0.081 ± 0.00	0.055 ± 0.00	0.030 ± 0.00
	pH	6.20 ± 0.10	5.8 ± 0.13	5.62 ± 0.16	5.2 ± 0.12

Data represented as mean ± SD, *n* = 3.

### 3.6. Storage stability

In this study, the optimized NAR-ME was stored at 4 °C, 25 °C, and 40 °C to evaluate its physical and chemical stability. During storage at all three temperatures for a total of 3 months, NAR-ME remained transparent and did not show any phase separation, turbidity, or precipitation, indicating that the optimized NAR-ME demonstrated excellent thermodynamic stability under long-term storage and accelerated conditions (40 °C). Moreover, there was no significant difference in drug content, DS or pH, which confirmed that Nar-ME had good chemical stability (Table 6).

### 3.7. *In vitro* human corneal epithelial cytotoxicity

**Figure 6.**
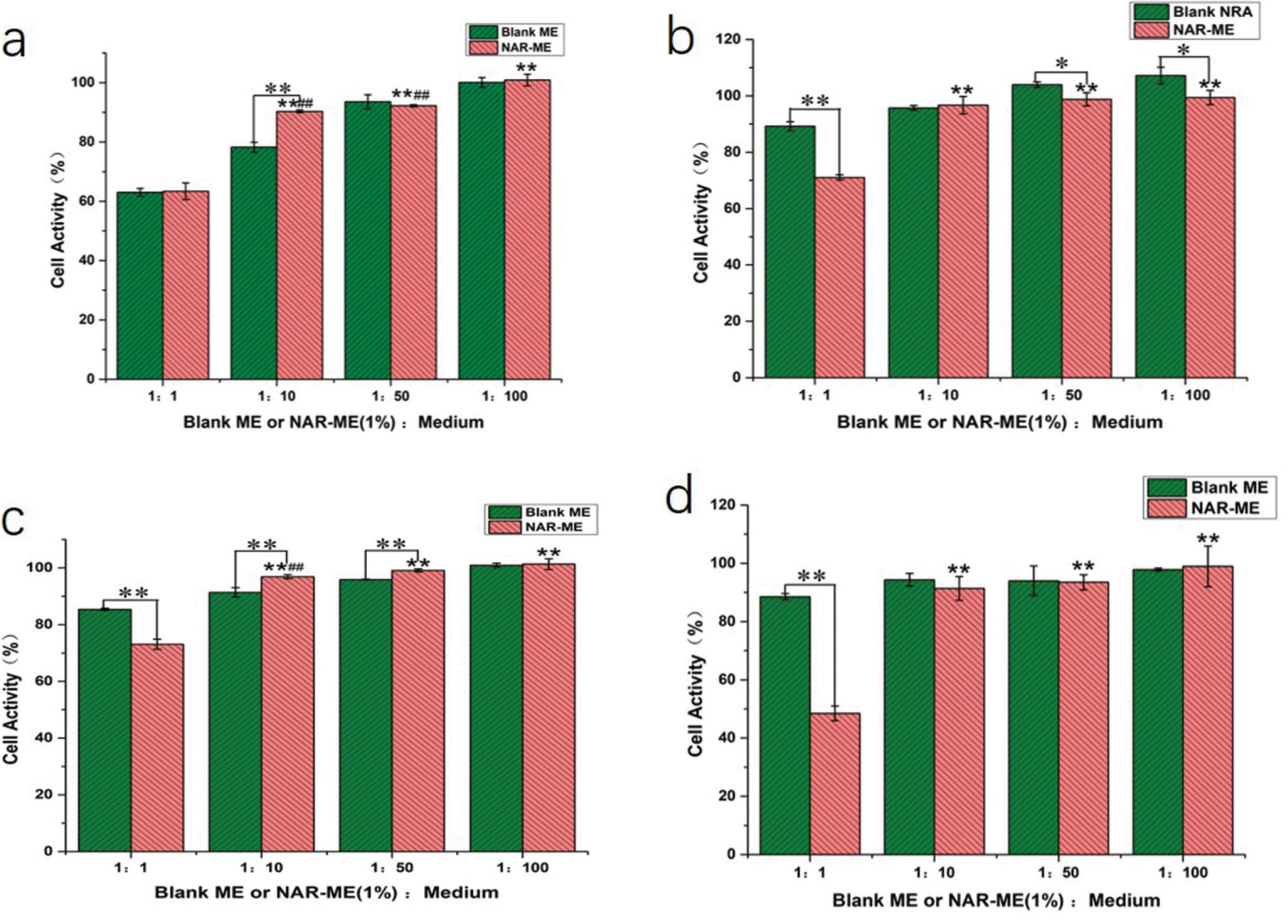
Cell activity of blank-ME and NAR-ME at different time points: (a) 0.25 h; (b) 1 h; (c) 2 h; (d) 4 h. Data represented as mean ± SEM, *n* = 6. *p<.05, **p<.01. Independent samples *t*-test. *p<.05, **p<.01 vs. 1:1 NAR-ME; ^##^*p*<.01 vs. 1:100 NAR-ME. One-way ANOVA, followed by Fisher’s least significant difference (LSD).

To confirm the *in vitro* safety of the materials, HCECs were used to research the cytotoxicity of NAR-ME by CCK-8 assay. The cell viability of HCECs was measured after incubation with various concentrations of blank ME and corresponding NAR-loaded formulations for different times (0.25, 1, 2, and 4 h). As shown in Figure 6, NAR-ME did not show any cytotoxicity against HCECs at final concentrations ranging from 0.1 mg/mL to 5 mg/mL. These results suggested that the viability of these cells is not affected by NAR-ME.

### 3.8. Ocular irritation test

**Figure 7.**
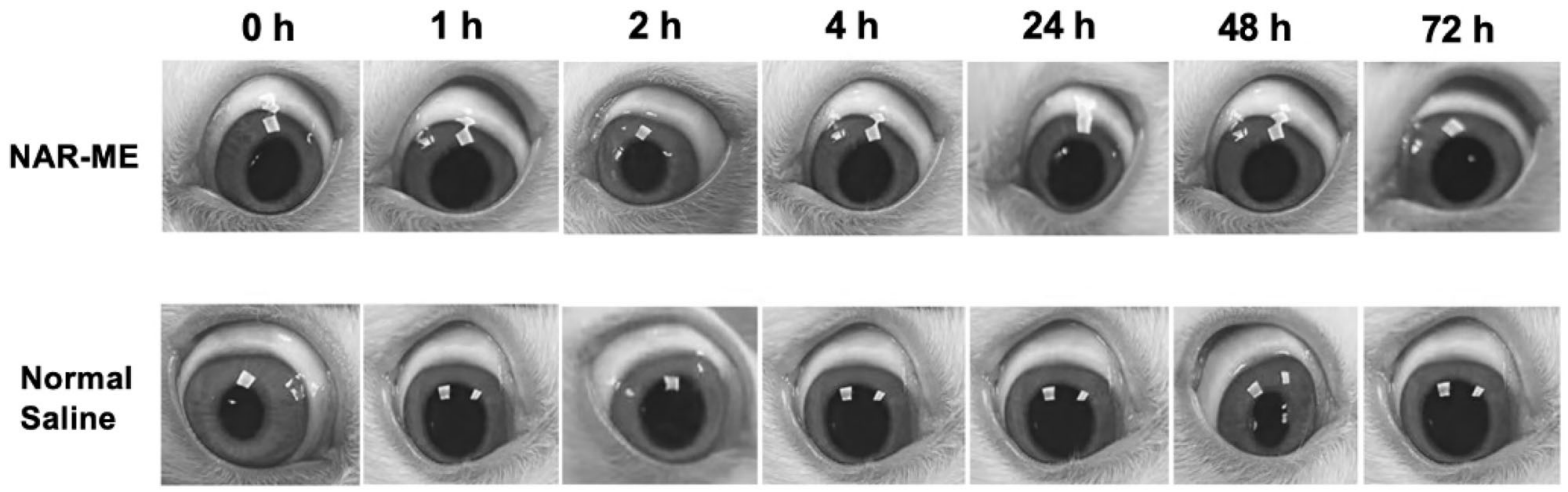
Local ocular reaction observed under slit lamp microscope after *in vivo* single instillation of 0.5% NAR-ME or normal saline (served as control).

The Draize test results revealed no sign of ocular damage or clinical abnormalities in the conjunctiva, cornea, iris, or pupil region, and the corresponding grading scores of the symptom signs were 0 for saline and 1% NAR-ME (Figure 7). All these results indicated that 1% NAR-ME is safe and causes no ocular irritation.

### 3.9. *Ex vivo* corneal permeation study

**Figure 8.**
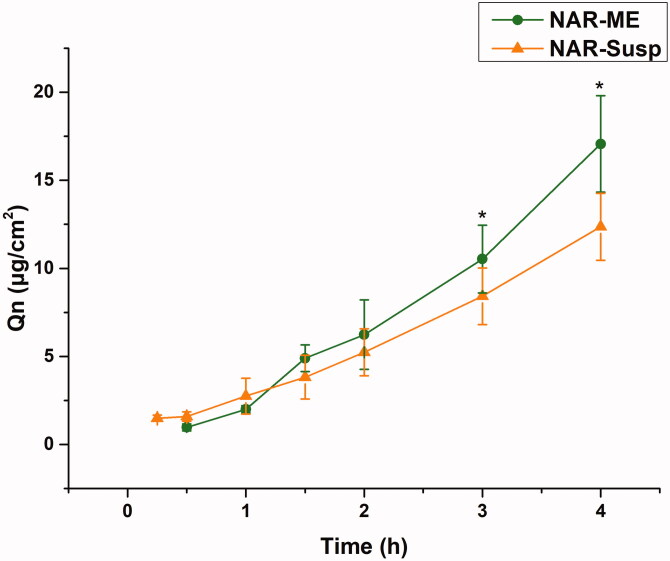
Cumulative corneal permeation profiles of the ME and Susp solution. Data represented as mean ± SEM, *n* = 6. *p<.05. Independent samples *t*-test.

The corneal permeability of a drug is the key to determining the pharmaceutical effect, and it is also the main factor determining the bioavailability of ophthalmic preparations. The corneal permeation parameters are listed in Table 7. All data are the mean of three determinations. The *P_app_* and *J* values of the NAR-ME group were significantly higher than those of the NAR-Susp group (*p*< .05). The results indicated that the ME promoted corneal penetration of NAR. The HL is a sensitive index to measure corneal integrity in *in vitro* experiments, and the degree of hydration has an enormous influence on drug penetration. Both the HL of the cornea of the two preparations did not exceed 83%, indicating that there was no damage to the epithelial and endothelial cells of the cornea during the experiment, and the samples caused no obvious irritation to the cornea (Figure 8).

**Table 7.** Permeability coefficients and hydration levels of the ME and Susp solution.

Formulae	Permeability coefficients		
	*P_app_*×10^–8^ (cm/s)	*J* × 10^–4^ (μg/cm^2^/s)	HL (%)
NAR-ME	31.95 ± 7.12	12.78 ± 2.85	80.76 ± 0.57
NAR-Susp	20.13 ± 4.68	8.05 ± 1.87	82.61 ± 0.27

Data represented as mean ± SD, *n* = 6.

### 3.10. Ocular pharmacokinetic studies in rabbits

**Figure 9.**
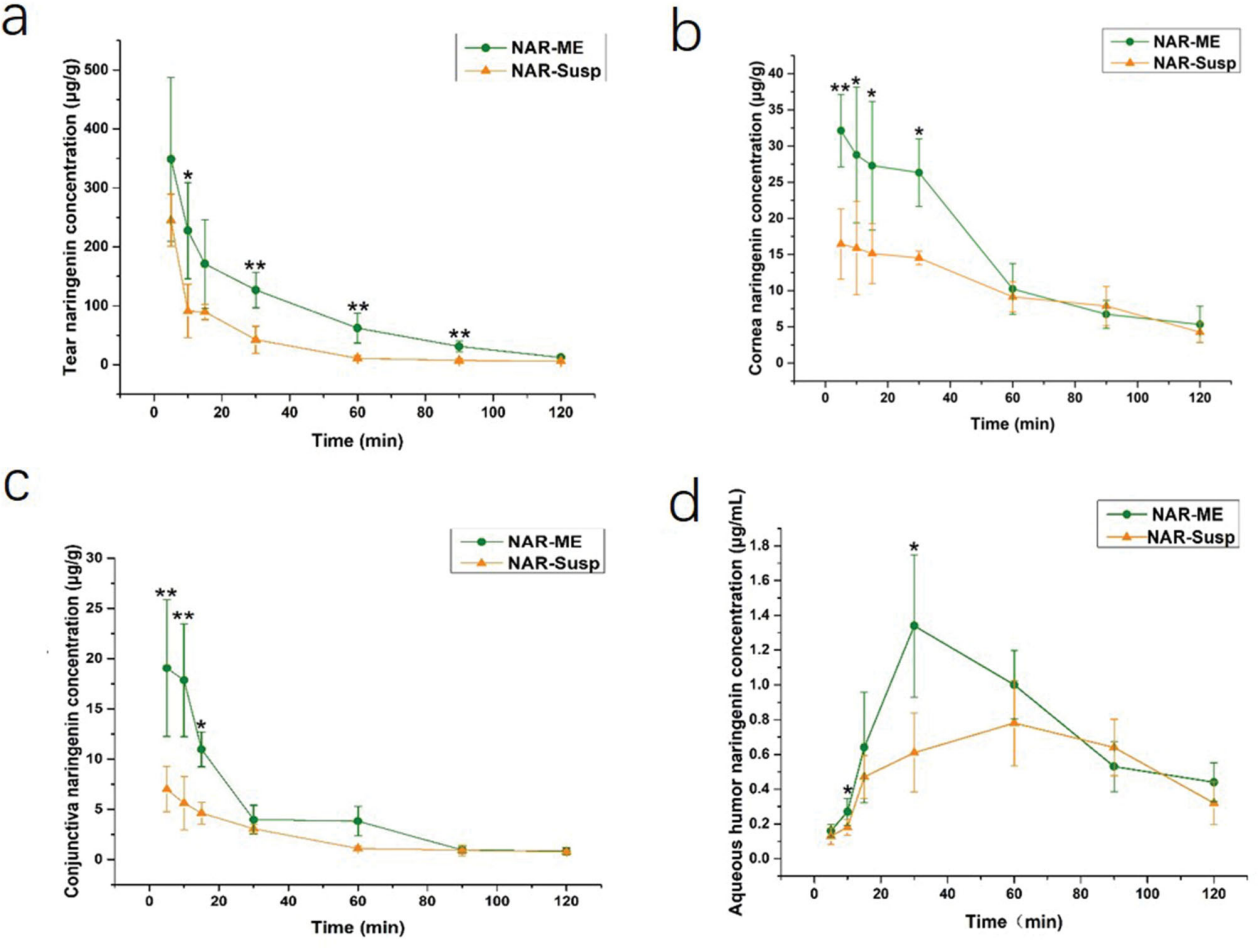
The concentration–time profiles of the ME and Susp solution: (a) tear; (b) cornea; (c) conjunctiva; (d) aqueous humor. Data represented as mean ± SEM, *n* = 6. *p<.05, **p<.01. Independent samples *t*-test.

The ocular distributions of NAR in the tears, cornea, conjunctiva, and aqueous humor following topical administration of NAR-ME and NAR-Susp are shown in Figure 9. Significantly higher concentrations of NAR were observed in these tissues following treatment with NAR-ME than treatment with NAR-Susp, especially at 10 and 30 min after instillation. The NAR levels in the tears, cornea, conjunctiva, and aqueous humor observed following instillation of NAR-ME were 2.50-, 1.81-, 3.18-, and 1.5-fold higher, respectively, at 10 min and 2.99-, 1.81-, 1.30-, and 2.20-fold higher at 30 min than those observed in the same sites following the use of NAR-Susp. Ocular pharmacokinetics also revealed a 1.45-fold, 2.15-fold and 1.35-fold increase in the area under concentration–time curves (AUC_0-120_ _min_) over 120 min in cornea, conjunctiva, and aqueous humor, respectively, with NAR-ME compared to NAR-Susp in rabbits after single-dose administration (Table 8).

**Table 8.** Pharmacokinetic parameters following topical application in rabbits.

Tissue	Pharmacokinetic parameters	Unit	NAR-ME	NAR-Susp
Tear	*C*_max_	μg/g	348.82 ± 159.01	245.28 ± 48.06
	*T*_max_	min	5	5
	*T*_1/2_	min	21.364	29.542
	ACU_0–_*_t_*	mg/L*min	10671.429	6068.837
	MRT_0–_*_t_*	min	17.424	32.487
Cornea	*C*_max_	μg/g	32.12 ± 2.17	16.45 ± 3.31
	*T*_max_	min	5	5
	*T*_1/2_	min	35.689	57.24
	ACU_0–_*_t_*	mg/L*min	1758.95	1214.775
	MRT_0–_*_t_*	min	39.991	48.32
Conjunctiva	*C*_max_	μg/g	19.07 ± 7.01	6.99 ± 2.02
	*T*_max_	min	5	5
	*T*_1/2_	min	28.406	112.455
	ACU_0–_*_t_*	mg/L*min	539.475	250.35
	MRT_0–_*_t_*	min	30.892	36.24
Aqueous humor	*C*_max_	μg/g	1.34 ± 0.46	0.78 ± 0.24
	*T*_max_	min	30	60
	*T*_1/2_	min	55.171	46.668
	ACU_0–_*_t_*	mg/L*min	91.2	67.375
	MRT_0–_*_t_*	min	55.274	62.375

*C*_max_: maximum concentration; *T*_max_: time of maximum concentration; *T*_1/2_: elimination half-life; AUC_0–_*_t_*: area under the concentration–time curve between 0 and 2 h; MRT_0–_*_t_*: mean residence time between 0 and 2 h.

Data represented as mean ± SEM, *n* = 6.

### 3.11. *In vivo* anti-corneal neovascularization efficacy

#### 3.11.1. Observation and quantification of CNV

**Figure 10.**
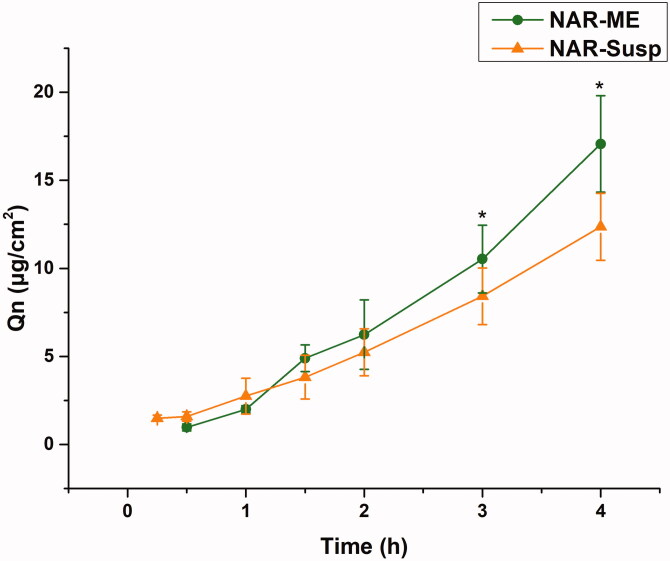
The area of corneal epithelial injury in mice at day 0 post-alkali burn.

**Figure 11.**
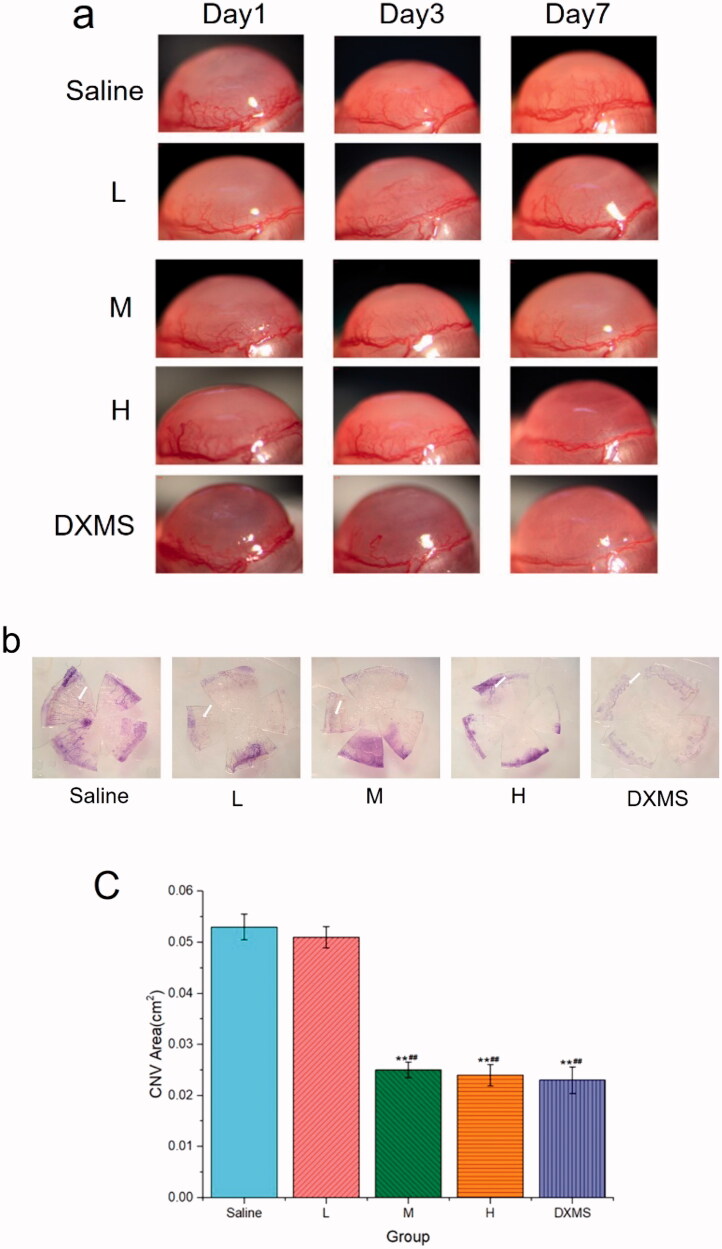
Area of corneal neovascularization: (a) observation of corneal neovascularization (CNV) area in the different group with slit lamp at different time points; (b) CNV area at seven days after alkali burn by hematoxylin perfusion (the white arrow indicates); (c) area of corneal neovascularization at seven days after alkali burn. Data represent the mean ± SEM of three mice per group (**p<.01 vs. saline; ^##^*p*<.01 vs. L. one-way ANOVA, followed by Fisher’s least significant difference (LSD)).

To investigate the anti-CNV effects of NAR-ME, an alkali-induced CNV mouse model was established. As shown in Figure 10, fluorescent sodium staining of the corneal epithelium showed that the defect area of the corneal epithelium was similar in each group, which indicated that the model was built successfully, and there was no significant difference between each group. To investigate whether daily treatment with NAR-ME would reduce CNV, corneal images (Figure 11(a)) were obtained with a slit lamp microscope system. Alkali burn injury induced a significant increase in the area of neovascularization in the cornea at 3 and 7 days (Figure 11(a)) with saline treatment. Daily treatment with 100 μg and 200 μg but not lower doses (50 μg) of NAR significantly reduced the area of neovascularization induced by alkali injury at seven days (Figure 11(b,c)). This treatment was even comparable to that with DXMS. NAR (50 μg) alone did not reduce the neovascularization area. The neovascularization area percentage was significantly higher in the saline and L groups than in the other groups (*p*< .01) (Figure 11(b)). The neovascularization area was significantly smaller in the M, H, and DXMS groups than in the saline group (*p*< .05). There was no statistically significant difference when compared to groups M, H, and DXMS (*p*> .05). These results prove the ability of NAR to reduce neovascularization in the cornea.

#### 3.11.2. Histopathological examination

**Figure 12.**
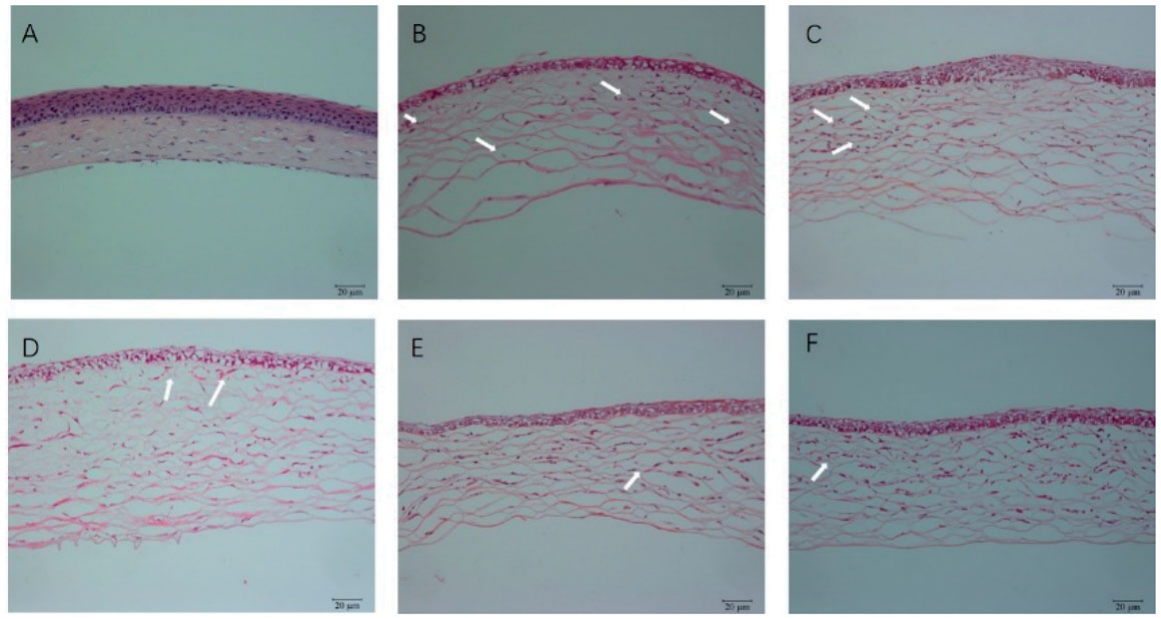
HE staining of cornea: (A) hematoxylin and eosin (HE) staining of corneal sections in the normal group (×200); (B) HE staining of corneal sections in the saline group (×200); (C) HE staining of corneal sections in the 0.25% NAR-ME group (L group) (×200); (D) HE staining of corneal sections in the 0.5% NAR-ME group (M group) (×200); (E) HE staining of corneal sections in the 1% NAR-ME group (H group) (×200); (F) HE staining of corneal sections in the glucocorticoid group (DXMS group) (×200). The white arrow indicates corneal neovascularization.

Hematoxylin and eosin-stained corneal sections were used to estimate the structural differences in different groups. Normal corneas revealed neatly arranged epithelial cells, regularly arrayed collagen fibers in the stroma and no vascular structure or pathological changes, as shown in Figure 12(A). The high-concentration NAR-ME group (Figure 12(E)) and medium-concentration NAR-ME group (Figure 12(D)) showed no significant changes compared with the DXMS group (Figure 12(F)). For the control group (alkali burn injury treated with normal saline) (Figure 12(B)) and low-concentration NAR-ME group (Figure 12(C)), a significant decrease in corneal epithelium thickness, irregular arrangement of epithelial cells, thickened collagen fiber spaces, disordered collagen fiber arrangement in the stroma, and neovascularization in the superficial stroma were observed. These results were in agreement with the results of the CNV area.

#### 3.11.3. Enzyme-linked immunosorbent assay

**Figure 13.**
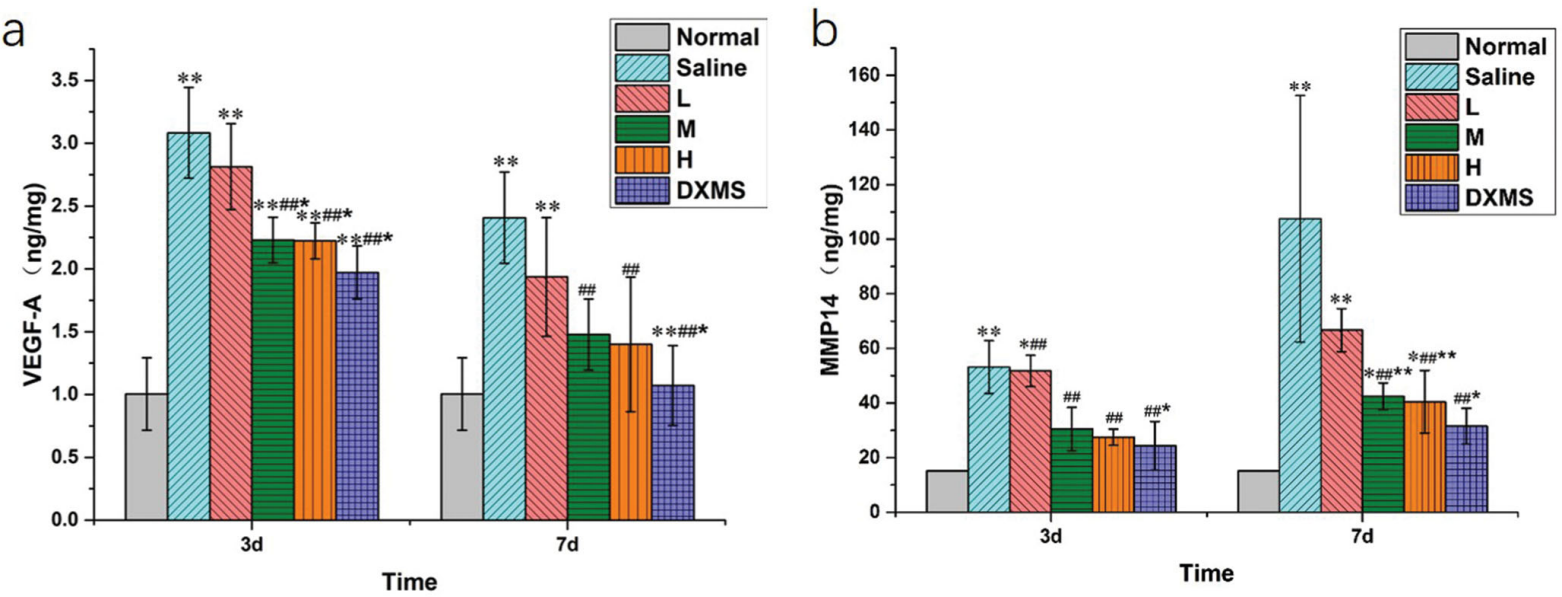
Protein expression in cornea at day 3 and day 7 post-alkali burn: the levels of VEGF-A (a) and MMP14 (b) were determined by Elisa in the cornea tissue 3 and 7 days after alkaline-induced cornea burn, respectively. Data represent the mean ± SEM of five mice per group (*p<.05, **p<.01 vs. normal; ^##^*p*<.01 vs. saline; and *p<.05, **p<.01 vs. L. one-way ANOVA, followed by Fisher’s least significant difference (LSD).

To analyze the effect of NAR-ME on protein expression in CNV, we measured the levels of VEGF-A and MMP14 in the cornea by ELISA. At both 3 days and 7 days, compared with those in the normal group, the VEGF-A and MMP14 levels were found to be significantly increased in the saline group (*p*> .01), whereas NAR-ME (M and H) and DXMS treatment prevented VEGF-A and MMP14 production (*p*> .05). These results suggested that NAR could inhibit VEGF-A and MMP14 expression in alkali-induced CNV mice (Figure 13).

## 4. Discussion

Regarding CNV, topical NAR was demonstrated to be effective as an antiangiogenic agent after alkali injury in experimental animal models (Oguido et al., 2017). However, it is well known that NAR has poor water solubility and low bioavailability (Hu et al., 2021). In terms of improving the bioavailability of NAR and achieving better therapeutic effects, MEs as topical ocular carriers can lead to great ocular drug absorption. In the present study, we successfully prepared a novel O/W NAR-ME and estimated its *in vitro* and *in vivo* performance characteristics.

The ME undoubtedly enhanced the ocular bioavailability of insoluble drugs. All selected vehicles contained no organic solvents, overcoming toxicity issues caused by organic residues. These vehicles are safe and harmless for ophthalmic preparations. An O/W formulation using a 4:1 CRH40:PEG400 ratio resulted in the maximum range of ME areas. The response surface plots demonstrating the role of independent variables on DS and DL are illustrated in Figure 2. The model terms *A*, *B*, *A*^2^, *B*^2^, and *AB* were significant model terms (*p*< .05), with *A* and *B* as independent variables and *AB* as the interaction term of the independent variables. The positive and negative effect signs represent the positive and negative effects on the response. The equation showed that an increase in oil weight (*A*) resulted in an increase in DS and a decrease in DL. According to the relevant literature, the stability of MEs is related to the DS of the emulsion, which means that a smaller DS can avoid aggregation and precipitation and thus result in better stability (Zeng et al., 2021). The characteristics of small DS and PDI, high DL and EE can also enhance the permeability. Optimized formulations are very easy to prepare because their pH and osmotic pressure have met the requirements of ophthalmic preparations without adjustment. Drug release plays a significant role in the fate of MEs *in vitro*, as only the drug released from the delivery system exerts a pharmacologic effect (Su et al., 2017). Generally, the *in vitro* release curve of PBS is closest to that of ocular release. NAR-ME and NAR-Susp were immersed in a system of a fixed volume (100 mL) and in a closed and relatively static environment, respectively. However, while *in vitro* release tests can provide important parameters for *in vivo* research to a certain extent, they can never replace *in vivo* tests (Han et al., 2021). The drug release curves were fitted to different models, and for NAR-ME, the correlation coefficient *R*^2^ of the Higuchi release model was the highest. The cumulative release of NAR from the ME was significantly higher than that from Susp after 24 h. After 72 h, the release of NAR from the ME reached 80%, which means that MEs release faster and have better release properties. The results of the stability experiment confirmed the developed optimal formulation, which can thus be properly used and stored．

It is well known that corneal epithelial damage caused by eye drops can lead to corneal opacity. Therefore, it is necessary to study the toxicity of eye drops on corneal epithelial cells. In our study, the cytotoxic properties of O/W NAR-ME and blank ME were assessed in the HCECs. Both treatments were considered nearly noncytotoxic. The reason behind the safety of the ME formulation might be related to its prescription ingredients, and usually, the retention time of eye preparations in the cornea is not very long. However, past studies have shown that NAR is slightly toxic to the corneal epithelium with the extension of culture time (Guan et al., 2021). Both 1:1 NAR-ME diluted with medium and blank ME showed obvious cytotoxicity in 15 min but gradually improved after an hour. This result may be due to the great changes in the environment of the cells before adding CCK-8, resulting in a stress response of the cells. The toxicity of the ME supplemented with naringin was less than that of the blank ME, which may be due to the antioxidative stress ability of naringin. At the same time, our experiment confirmed that the 1% NAR ME caused no irritation to rabbit eyes. The reason behind such compatibility of the ME formulation with the eye might be attributed to its NAR content, which provides a great anti-inflammatory effect, oxidative stress resistance, and immunoregulation effect (Francis et al., 1989; Chang et al., 2021; El-Wafaey et al., 2021; Miles & Calder, 2021; Sun et al., 2021).

Corneal penetration is an important rate-limiting step in the bioavailability of topical ophthalmic formulations that incorporate poorly permeable drugs. The optimized ME formulation exhibited better corneal permeation parameters than the Susp. The corneal permeability of the ME increased by approximately 1.6-fold. This result is mainly due to the ME increasing the solubility of NAR. On the other hand, due to the small size of the ME, it can easily enter epithelial cells and enhance absorption. The corneal hydration value is a subtle indicator of corneal tissue irritability *in vitro* (Chen et al., 2012). In this study, the HL values of corneas treated with NAR-ME and NAR-Susp did not exceed 83%. Therefore, neither NAR-ME nor NAR-Susp had significant corneal irritation effects.

Ocular pharmacokinetics has advantages and limitations, and it can directly determine the content of drugs in the target tissue. However, since the collection of tissues such as cornea and conjunctiva is irreversible, it is impossible to collect biological samples of the same experimental object at different time points. Therefore, the measured drug concentration is affected by individual absorption and metabolism to varying degrees (Zhou et al., 2020). Fortunately, our experimental results showed that the individual differences in naringin were very small. Compared to NAR-Susp, NAR-ME was rapidly dispersed in the cornea, conjunctiva, and aqueous humor to form an ME with a DS less than 20 nm, which could penetrate the absorption site via the transcellular pathway. In addition to the high solubilization effect, nonionic surfactants may decrease the interfacial surface tension and increase the penetration of the drug through epithelial cells (Craig et al., 1995; Rahman et al., 2013).

The results showed that topical administration of 0.5% NAR-ME significantly reduced the levels of VEGF-A and MMP14 in cornea tissue, and the CNV area was also greatly reduced. Numerous reports have shown that NAR can inhibit VEGF-A-induced angiogenesis through different pathways (Li et al., 2016; Dmytrenko et al., 2017; Pafumi et al., 2017). A similar observation has been documented by Oguido et al. (2017). MMP14 has been implicated in angiogenesis as not only a proangiogenic factor but also an antiangiogenic factor, and both up- and downregulation of MMP14 has been observed in a variety of disease models of angiogenesis. However, MMP14 is generally regarded as proangiogenic because it is upregulated during angiogenesis in endothelial tissue, and deficiency of MMP14 leads to a loss of the ability of endothelial cells to form new vessels (Han et al., 2015, 2019). Thus, NAR-ME may inhibit CNV by reducing the expression of proangiogenic growth factors such as VEGF-A and MMP14.

Our experiments showed that the NAR-ME improved the bioavailability of NAR in the eyes and has a significant inhibitory effect on CNV, representing an alternative to DXMS as an anti-CNV agent. Based on our findings, it remains puzzling why both 1:1 NAR-ME and blank ME were cytotoxic at 15 min. Further studies are needed to confirm the oxidative stress reaction in cytotoxicity experiments.

## 5. Conclusions

An optimized NAR-ME was prepared by using CCD method, and the obtained ME had good dilution stability and storage stability. Compared with that of the NAR-Susp, the *in vitro* release of the ME was significantly increased, and the cumulative absorption amount of the cornea was increased. *In vivo* pharmacokinetics showed that the ME increased drug absorption in the cornea by 1.95-fold. In summary, ME might increase drug bioavailability by increasing drug solubility and improving drug corneal permeability, with no obvious toxicity against HCECs. More importantly, MEs are relatively easily administered and safe for continuous delivery of NAR, leading to high anti-VEGF and MMP-14 levels to inhibit CNV. Therefore, NAR-ME is an ideal ocular delivery system for inhibition and a prospective treatment strategy for CNV.

## Data Availability

The raw/processed data required to reproduce these findings cannot be shared at this time as the data also forms part of an ongoing study.
